# Biomimetic Modification of Water-Borne Polymer Coating with Carnauba Wax for Controlled Release of Urea

**DOI:** 10.3390/ijms23137422

**Published:** 2022-07-04

**Authors:** Cong Ge, Xuebin Xu, Fei Ma, Jianmin Zhou, Changwen Du

**Affiliations:** 1 The State Key Laboratory of Soil and Sustainable Agriculture, Institute of Soil Science Chinese Academy of Sciences, Nanjing 210008, China; gecong20@mails.ucas.ac.cn (C.G.); xuxuebin@issas.ac.cn (X.X.); fma@issas.ac.cn (F.M.); jmzhou@issas.ac.cn (J.Z.); 2 College of Advanced Agricultural Sciences, University of Chinese Academy of Sciences, Beijing 100049, China

**Keywords:** carnauba wax, controlled release fertilizer, polyacrylate, biomimetic modification

## Abstract

Benefitting from the special structure of the leaf cuticle layer, plants have natural hydrophobicity and anti-fouling abilities. Inspired by the leaf surface structure, a biomimetic modification strategy was raised to improve the surface hydrophobicity of polyacrylate coating for controlled release fertilizer. Double-layer (polyacrylate and carnauba wax) coated fertilizer was obtained after biomimetic modification. The quality of controlled release fertilizer modified with the carnauba wax was greatly enhanced, and the coating material was effectively saved. The surface appearance of polyacrylate-coated fertilizer was improved for the surface blemish was repaired by the loaded carnauba wax. The characterizations by Fourier transform infrared spectroscopy indicated that the hydrogen bonds were formed between the water-based polyacrylate membrane and the carnauba wax layers. By optimizing the content of polyacrylate and carnauba wax, the release duration of the fertilizer was effectively prolonged, which was improved from 1 month to more than 2 months after the biomimetic modification. Therefore, biological wax as an environmentally-friendly natural material that has showed a broad potential in the application of coated controlled release fertilizer.

## 1. Introduction

Chemical fertilizer plays an important role in maintaining crop growth and ensuring food security [1,2]. However, the low utilization rate of chemical fertilizer is an important challenge for food production at this stage, which will lead to environmental pollution, global climate warming, waste of resources, and other problems that harm human health and development [3,4,5,6]. Controlled release fertilizer (CRF) encapsulated by a polymer can significantly improve the release duration of nutrients, and thereby manage nutrients accurately, improve the use efficiency, and reduce adverse impacts on the environment [7,8]. Due to the sustainability and predictability of the release, polymer-coated CRF is widely used for delaying or controlling the release of nutrients to preferably match the uptake dynamics of crops [9]. However, most of the traditional polymer coating materials are derived from petroleum and required organic solvents throughout the production process, so they might be expensive, non-renewable, and harmful to the environment, which makes them unsuitable for use in agricultural production [10,11,12].

Waterborne polyacrylate (WPA) is a new type of organic polymer coating material prepared by using water as a solvent. WPA has excellent film-forming properties, appropriate viscosity, and low price [13]. As an environment-friendly material, it barely harms the soil structure, function, and microbial activities [14]. However, the hydroxyl and carboxyl groups of aqueous polyacrylates make the polymer hydrophilic and easy to absorb water and expand, resulting in excessive nutrients released in the first few days and shortening the overall release longevity [15]. To improve the hydrophobicity, Shen et al., (2015) applied aziridine to modify the WPA [16]. The addition of aziridine reduced the content of the carboxyl group in WPA and improved the water resistance of the membrane. Shen et al. (2014) further employed microemulsion polymerization technique to change aqueous polyacrylate properties, which strengthened the water-resistance performance, and reached the purpose with a higher glass-transition temperature and a strain-hardening effect [15]. However, the implementation of these strategies not only increased the cost and made the preparation steps more cumbersome, but also the controlled release performance showed potential to be further improved.

Natural plants, such as lotus leaf [17,18], rice leaf [19,20], and rose petal [21], have a specially textured topography of the surface and the chemical constituency of the cuticle, which is composed of soluble lipids embedded in a polyester matrix, covering their surface [22]. Many studies have revealed that this texture structure, particularly due to epicuticular wax crystals, is the structural basis of exceptional water-repellency of surfaces [23,24]. The hydrophobic surface provides waterproof and self-cleaning functions for plants [25,26], which provides a novel strategy for preparing CRF that employs biological wax to modify the physical and chemical properties of the polyacrylate coating surface for polyacrylate-coated fertilizer. Biological wax is a mixture of hydrophobic compounds derived from plant cuticles, seed coatings, or insects’ secretion [27], and Carnauba wax (CW), derived from the leaves of the Copernicus tree [28], not only has strong hydrophobic ability, but also has good performance in adhesion and friction resistance compared with other biological waxes. It is mainly composed of about 80% long-chain wax esters and the remaining 20% is composed of fatty acids, fatty alcohols, and hydrocarbons [29,30]. It is widely used in various industrial applications, and also has a good performance in the encapsulation and controlled release of drugs [31,32]. The modification by CW considerably improved the material’s hydrophobicity and water-tightness, and the technique was quite straightforward. Furthermore, CW is an environment-friendly material that is biodegradable and has no impact on the environment [33]. Therefore, biomimetic modification by CW has broad prospects in improving the controlled release longevity of waterborne polyacrylate -coated fertilizer and reducing the cost of coating material.

In this study, inspired by the structure of plant cuticle layer, which contains wax-polymer complexes, natural CW was applied to modify the surface of waterborne polyacrylate coated urea (PACU). The superhydrophobic coated urea that simultaneously combines natural bio-safety and long controlled release longevity was synthesized by optimizing the proportion of WPA coating rate and CW loading amount. The surface appearance and chemical composition of the modified coating were characterized by the scanning electron microscope, Fourier transforms infrared spectroscopy, and laser-induced breakdown spectroscopy. The nutrient release performance was determined using water incubation in the laboratory. The main objectives of this study are: (i) to develop a novel biomimetic modification for CRF; (ii) to explore the interfacial adhesion mechanism between CW and WPA; and (iii) to improve the nutrient release performance of waterborne polyacrylate- coated CRF.

## 2. Results

### 2.1. Optimization of Carnauba Wax Modefication

**Figure 1:**
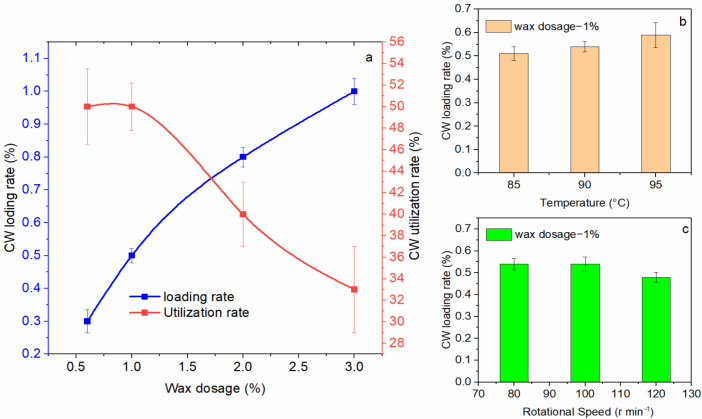
Factors affecting the carnauba wax (CW) loading rate. (**a**) Dosage; (**b**) Temperature; (**c**) Rotational speed.

The CW dosage played a key role in loading rate, and the CW loading rate increased with the raised dosage, but the utilization rate decreased on the contrary (Figure 1a). Presumably, there was a limit to the amount of wax loading on the PACU surface. When the CW formed a continuous film on the fertilizer surface, the load volume would be increased slightly or no increase. From Figure 1b it was found that temperature showed little effect on loading rate when it was between 85 °C and 95 °C. The temperature range was selected for the melting point of CW was 82 °C, and the urea volatilize would significantly occurred when the temperature was higher than 100 °C. It was observed that rotational speed also demonstrated little influence on the envelope volume in the range of 85 r min^−1^ to 125 r min^−1^. The Fertilizer granules would stay at the bottom of the flask when the rotational speed was below 80 r min^−1^, resulting an ununiform loading of wax on the fertilizer; on the other side, when rotational speed was higher than 130 r min^−1^, the fertilizer granules moved too fast and it would stick to the wall of the flask, which also resulted in ununiform wax loading.

### 2.2. Morphological Structure

**Figure 2:**
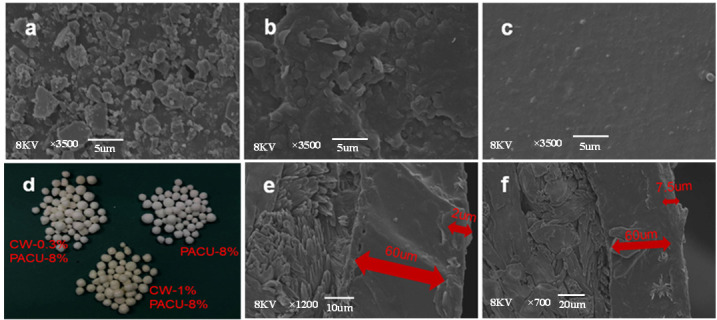
Scanning electron microscopy (SEM) images of the surface and section of coated urea. (**a**) Surface of PACU with 8% content of PWA. (**b**) Surface of CWPACU with 8% content of PWA and 0.3% content of Carnauba wax (CW). (**c**) Surface of CWPACU with 8% content of PWA and 1% content of CW. (**d**) appearance of coated urea using varied rate s of CW and PACU. (**e**) Section of CWPACU with 8% content of PWA and 0.3% content of CW. (**f**) Section of CWPACU with 8% content of PWA and 1% content of CW.

The CW modified PACU was developed as double-layer coated fertilizer, and the combination mode and morphological characteristics between the two layers of film wrapping the fertilizer core played key roles in nutrient release. The distinct interface between CW and WPA was observed from the SEM images (Figure 2), and it was seen that the fertilizer core was a granular crystal differentiated from the non-crystal coating. The thickness of WPA film was around 60 µm while the thickness of CW film was thinner as around 7.5 µm when the CW input was 1%, and the membrane layers adhered well to each other. When the same film material was used for multi-layer coating, slight dissolution would occur between the film layers, reducing the thickness of the film [34].

There were many residual polyacrylate particles on the surface of the water-based polymer coating. The internal WPA granules formed a compact polyacrylate membrane to control the flow of nutrients and water. However, these surface particles were only randomly distributed on the fertilizer surface and failed to form a continuous film. Therefore, they did not play a role in slowing down the release of nutrients, resulting in a waste of coating materials. Moreover, there were many micropores on the WPA membrane surface, which accelerated the entry of water into the membrane [35]. The CW modification integrated the scattered particles on the surface into a monolithic and compact membrane to make full use, and it also blocked the micropores on the surface of the polyacrylate film and reduced the membrane diffusion coefficient. Without CW loading there would generate many overlapped cracks on the surface of the coating shell, resulting in water entering the film from these cracks. When the CW loading increased, the WPA surface became continuous, uniform, and smooth, which contributed to improve the controlled release performance. In general, the loading rate of CW had a great influence on the surface morphology and the controlled release performance.

### 2.3. FTIR Characterization

**Figure 3:**
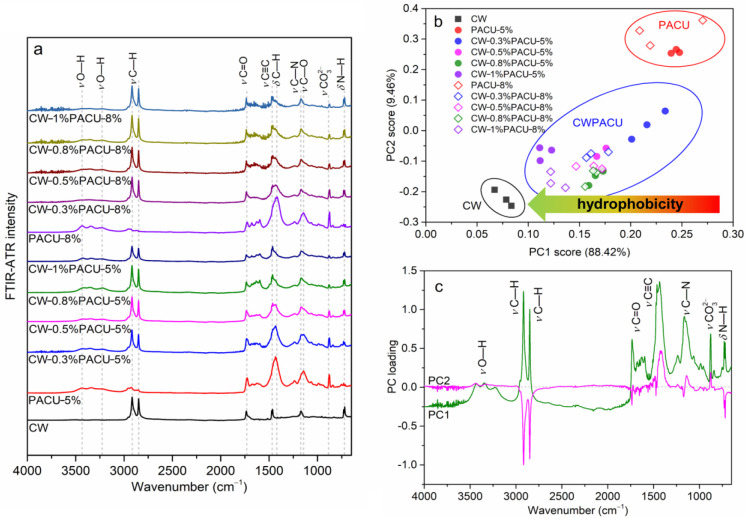
FTIR-ATR spectral characteristic of the PACU and CWPACU. (**a**) Comparison between the FTIR-ATR spectra of PACU and CWPACU. (**b**) Principal component scores of FTIR-ATR spectra for c PACU and CWPACU. (**c**) Principal component loading of FTIR-ATR spectra for different PACU and CWPACU.

The FTIR spectra of CW and PACU before and after CW modification were shown in Figure 3. The FTIR spectra featured a broad peak at ~3436 and ~3220 cm^−1^, which were attributed to the stretching vibration of O–H [36,37]. The shoulders peaks at ~2915 cm^−1^ and ~1848 cm^−1^ were dominated by the symmetrical and asymmetrical stretching vibration of C–H from methyl and methylene groups respectively [38]. The sharp peak at ~1730 cm^−1^ was associated with the stretching vibration of C=O from esters and diesters. The band at ~1468 cm^−1^ was dominated by the skeletal vibration of benzene. An obvious sharp peak at ~1515 cm^−1^ was attributed to the bending vibration (δ) of C–H from methyls. The peak at ~1161 cm^−1^ was confirmed as the stretching vibration of C–N from secondary amine. The peak at ~1143 cm^−1^ was dominated by the stretching vibration of C–O–C from the ether fatty chain. The out-of-plane bending vibration of N–H from secondary amine was confirmed at ~722 cm^−1^. In addition, the stretching vibration of carbonate was found at ~879 cm^−1^. The CW had obvious peaks of C–H, C=O, C≡C, C–N, and N–H, indicating that CW contained fatty acids, esters, fatty alcohols, aromatic and aliphatic hydrocarbons, and amide. Compared with the pure polyacrylate membrane, the shoulder peaks of C–H at ~2915 cm^−1^ and ~1848 cm^−1^ in the modified membrane were significantly higher, which suggested that the aliphatic group in the membrane was increased, and the aliphatic group was generally hydrophobic, thereby strengthening the hydrophobicity of the membrane.

The PACU showed similar peaks compared with CW but unique peaks of O–H and carbonate. The carbonate peak in PACU was caused by the addition of talcum powder in the process of fertilizer drying. After CW modification, the vibrations of O–H and carbonate decreased, and the vibrations of C–H and N–H increased, indicating the successful loading of CW onto PACU. With the increase of CW, the peak of C=O at ~1730 cm^−1^ from ester gradually decreased. The decrease of the ester group represented the decrease of the hydrophilic group, which would also increase the hydrophobicity of the membrane. Figure 3b,c show the PCA results of the FTIR spectra. The first principal component (PC1) accounted 88.42% of total variances, indicating that the PC1 could express the main variation of FTIR spectra. The PC scores of CW, CWPACU, and PACU were clustered into three clusters respectively. The CW and CWPACU had lower PC1 and PC2 scores than PACU. The PC1 and PC2 showed high loadings at the wavenumbers of the hydrophobic functional groups (C–H, C=O, C≡C, and C–N). These also indicated that the hydrophobic of the coated membrane was enhanced by CW modification.

**Figure 4:**
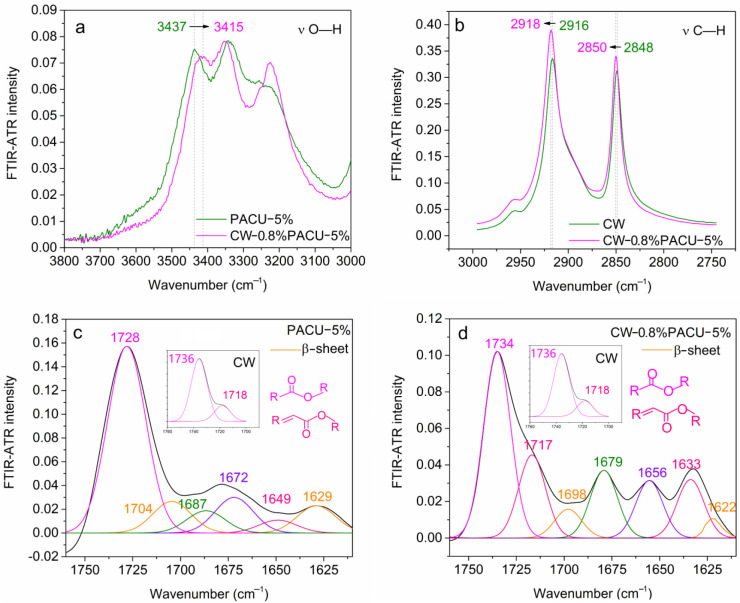
Changes in FTIR-ATR spectra after carnauba wax (CW) modification. (**a**) A redshift of the stretching vibration of O—H after CW modification. (**b**) A blueshift of the stretching vibration of C—H after CW modification. (**c**,**d**) Deconvolutions of the FTIR esters and diesters of PACU (**c**) and CWPACU (**d**).

The changes in some peaks’ positions between the PACU-5% and CW-0.8%/PACU-5% were further investigated to clarify the adhesion mechanism (Figure 4). Notably, the peak of O–H at 3437 cm^−1^ for the PACU was red-shifted to 3415 cm^−1^ for the CWPACU. The original β-sheet aggregate peaks in the PACU were observed at 1704 and 1629 cm^−1^. These two peaks were then clearly red-shifted to 1698 and 1622 cm^−1^, respectively, after CW modification. Furthermore, the C=O of –C=C–COOC– was also clearly red shifted from 1718 cm^−1^ in the CW to 1717 cm^−1^ for the CWPACU. The shoulder peaks of C–H at 2916 cm^−1^ and 2848 cm^−1^ for the CW were blue-shifted to 2918 cm^−1^ and 2850 cm^−1^ for the CWPACU. The red-shift of these FTIR peaks could be caused by the hydrogen bonds which would average the electron cloud density [39]. Hobza et al., (2000) summarized four blue shift hydrogen bond systems including the C–H [40]. Therefore, these results implied that hydrogen bonds were possibly formed between the WPA (O–H, C=O) and the CW layers (C–H, C=O).

### 2.4. LIBS Characterization

**Figure 5:**
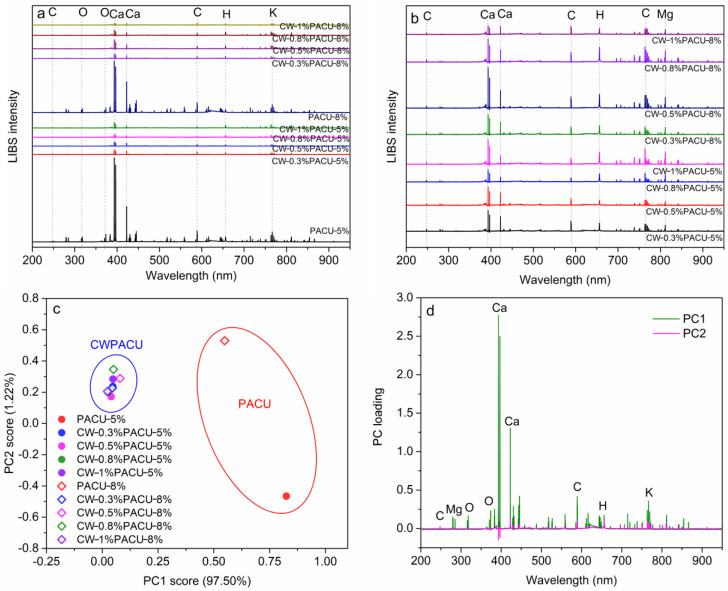
LIBS spectral characteristic of the PACU and CWPACU. (**a**) Comparison between the LIBS spectra of PACU and CWPACU. (**b**) Comparison between the LIBS spectra of CWPACU with different CW loading amounts. (**c**) Principal component scores of LIBS spectra for PACU and CWPACU. (**d**) Principal component loading of LIBS spectra for different PACU and CWPACU.

The LIBS spectra were compared with the NIST Atomic Spectrum Database to obtain the characteristic spectral lines of each element [41]. It was found that the increase in polyacrylate coating rate had no obvious effect on the change of substances (Figure 5a). The intensity of the spectral lines on the surface of CWPACU was much lower than that of PACU. The CW is a mixture of organics with different carbon chain lengths, with an average elastic chain length of 50 carbon atoms [42]. Therefore, the molecular weight was lower than that of synthetic polyacrylate, and the density was also lower, resulting in the low intensity of various spectral lines, which allowed the wax to degrade rapidly after the release of fertilizer without affecting the decomposition of polyacrylate. The spectral signals at 370.5 nm, 589.0 nm, and 656.2 nm were associated with O, C, and H, respectively. Since CW and polyacrylate were composed of these three elements, their proportion became the key factor determining their properties. The account for oxygen in CW was less than that in polyacrylate, which reflected that the content of the C–C and C–H in CW was high, while the content of the hydrogen–oxygen bond was less. With the increase in CW amount, the total content of C, H, and O increased, but the content of CII decreased and the CIII increased. It showed that the C–C increased and the C=C decreased, which improved the stability of the membrane and ensured its service life as the coating for nutrient controlled release. As a result, the coating would not degrade in the release process of fertilizer, resulting in the sudden release of fertilizer. Figure 4b,c show the PCA results of the LIBS spectra. The first principal component (PC1) accounted the 97.5% of total variances, indicating that the PC1 could express the main variation of LIBS spectra. The PC scores of CWPACU and PACU were clustered into two clusters, respectively. The CWPACU had a lower PC1 score than the PACU and higher loading at the wavelength of Ca lines, which was caused by the addition of talcum powder in the process of fertilizer drying.

### 2.5. Release Performance

**Figure 6:**
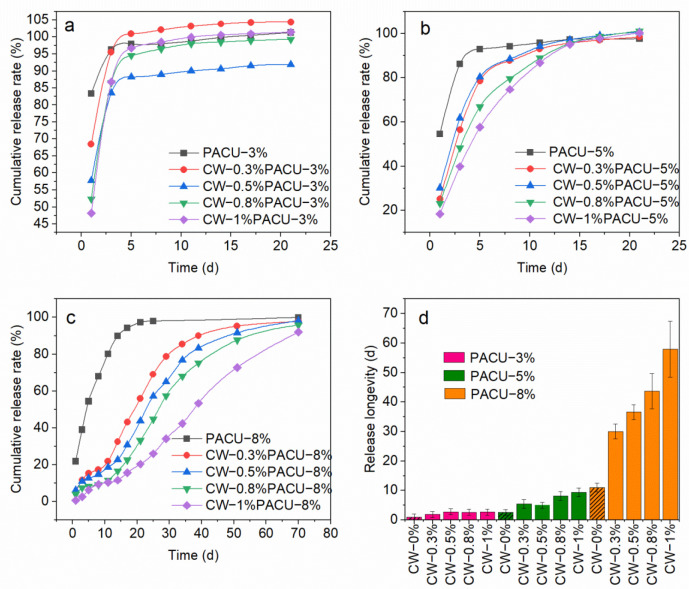
Release performance of coated urea with different Carnauba wax (CW) and WPA contents. (**a**) Urea coated with 3% WPA (PACU-3%). (**b**) Urea coated with 5% WPA (PACU-5%). (**c**) Urea coated with 8% WPA (PACU-8%). (**d**) Comparison of release longevity (80%) between different PACU and CWPACU.

Release longevity is an important criterion for evaluating the effectiveness of coating materials. In contrast to the PACU, biomimetic modification by CW greatly enhanced the release longevity. The release longevity of urea increased with the increase of CW loading rate and the coating rate of WPA. Figure 6a shows the release profile of CRF with a 3% WPA coating rate and different CW content. The 24 d cumulative release rate of urea for PACU-3% without loading CW was 83.3% while it was decreased to 68.4%, 57.8%, 52.4%, and 48.2%, respectively, by biomimetic modification with 0.3%, 0.5%, 0.8%, and 1.0% CW, respectively. As a result, the urea release longevities were extended from 24 h to 3 d. As the loading content of CW increased from 0% to 1%, the initial dissolution rate of PACU-5% decreased from 54.6% to 18.3% (Figure 6b). The release longevities also increased from 5 d to about 20 d, which was better to meet the nutrient demand of plants. In Figure 6c, the release longevity of PACU-8% was significantly improved from about 20 d to 60–80 d by biomimetic modification with CW. The 24 h cumulative release rate of urea for PACU-8% was decreased from 21.8% to 5.02%, 6.19%, 3.86%, and 0.64% by biomimetic modification with 0.3%, 0.5%, 0.8%, and 1.0% CW, respectively. The shape of the release curve for PACU-8% was transformed from “L” to “S” pattern [43], indicating changes in the release mechanism. Both CW loading rate and WPA coating rate had influences on the controlled-release performance of urea (Figure 6d). CW improved the hydrophobicity of the fertilizer surface and effectively prevents the entry of water in the early stage of fertilizer release. However, the toughness of the wax was poor. After the film absorbs water and swells, it was easy to cause the outer wax film to crack, resulting in the accelerated release of fertilizer. In general, the CWPACU had longer release longevity and a smoother release curve than PACU, which was better synchronized with the nutrient absorption of plants [44].

## 3. Discussion

**Figure 7:**
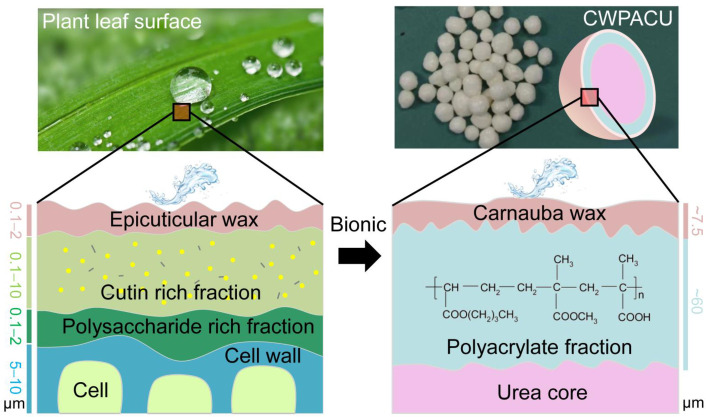
The carnauba wax-modified polyacrylate bionic double coating for controlled release fertilizer regarding the structure of plant leaf cuticle.

Plant cuticle covered the outside of epidermal cell is a thin layer, which is totally composed of two sublayers including epicuticular wax layer and hydrocarbon polymer layer, and the total layer thickness was proved to be around 0.1–10 μm (Figure 7) [45,46]. The cuticle layer can effectively control the flux of water and nutrients, which guaranteed the plant growth [47]. The point of coated fertilizer is the control of water and nutrient across the membrane, and thereof the structure of plant cuticle layer can be applied in fertilizer coating. Similarly, the polyacrylate functioned as hydrocarbon polymer layer (i.e., cutin, polysaccharides, etc.), and the carnauba wax functioned as epicuticular wax layer. The compatibility should be considered among sublayer, and for different wax and polymer the parameters related with the coating forming, such as temperature and coating rate, should be optimized. Fortunately, in this study, polyacrylate was well compatible with urea and wax, i.e., polyacrylate got well touched both with fertilizer core and Carnauba wax, so that the novel coating for controlled release of nutrient simulating plant cuticle layer was successfully developed.

**Figure 8:**
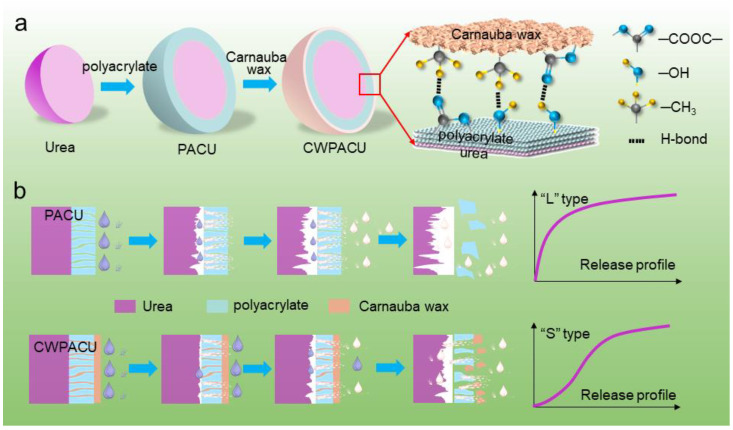
(**a**) Process of Carnauba wax (CW) biomimetic modification of PACU and film formation mechanism of CWPACU. (**b**) Release mechanism of the PACU and CWPACU.

The release longevity of urea was greatly prolonged under the combination treatments of CW and WPA. The PACU showed insufficient hydrophobicity and was easy to absorb water and swell, resulting in short release longevity of urea. The surface hydrophobicity of coated urea was improved by biomimetic modification of CW. The hydrogen bonds were possibly formed between the WPA membrane (O–H, C=O) and the CW layers (C–H, C=O) (Figure 8). This hydrophobic structure was beneficial to retarding water entry into the fertilizer core and enhancing the urea release longevity. In the initial release stage of CWPACU, the waxy layer on the surface reduced the contact between water and polyacrylate membrane. For the urea with WPA content of 3% and 5%, the release performance was still not satisfied even if it was modified by CW. The fundamental reason was that the coating rate of WPA was pretty low so that the water could still enter the fertilizer quickly. Moreover, the strength was also insufficient due to the low thinness of the film, and expansion and crack would easily occur (Figure 7). When the coating rate of WPA increased to 8%, urea could hardly be released at the initial stage. At this stage, the CW not only blocked the entry of water but also slowed down the swelling of WPA and prolonged the “lag period” [48] of urea release. In the later stage, restricted by the CW, the swelling degree of the membrane was still less than PACU, and the surface micropores would not swell significantly, which further prolonged the release duration of urea.

## 4. Materials and Methods

### 4.1. Materials

The CW in the form of flakes was purchased from Shanghai Macklin Biochemical Co., Ltd. (Shanghai, China) Methyl methacrylate (MMA, AR), n-butyl acrylate (BA, CP), methacrylic acid (MAA, CP), and urea (AR) were purchased from Nanjing Chemical Reagent Co., Ltd., Nanjing, China. Sodium dodecylbenzene sulfonate (SDBS, AR) was obtained from Chengdu Kelong Chemical Reagent Co., Ltd., Chengdu, China. Potassium persulfate (KPS; Chemical pure) was supplied by Sinopharm Chemical Reagent Co., Ltd. (Shanghai, China) Nonyl phenyl polyoxyethylene ether-10 (OP-10, CP) was obtained from Hebei Xingtai Kewang Auxiliary Agent Co., Ltd., Xingtai, China. The urea granules (46.6 wt.% of nitrogen and 2.00–4.75 mm in diameter) were purchased by Shandong Luxi Fertilizer Co., Ltd., Liaocheng, China.

### 4.2. Preparation of Polyacrylate Emulsion

The polyacrylate emulsion was prepared by a semi-continuous conventional emulsion polymerization procedure, and the full water uptake rate of the polyacrylate coating was around 15% [15]. The aqueous phase was prepared by the dissolution of 8.24 g of OP-10 and 4.12 g of SDBS in 248 g of water in a three-neck flask. Then, 110 g of BA, 90 g of MMA, and 5 g of MAA were mixed to form an oil phase. The oil phase was added to the aqueous phase and stirred vigorously for 30 min. The 75% emulsion was then poured out of the three-neck flask and the remaining was heated to increase the temperature to 85 °C. The initiator solution (50 mL, 0.013 g mL^−1^ KPS) and the mixture poured out of the three-neck flask were divided into four parts, alternately added in turn, and the whole process maintained the stirring rate of 200 rpm. After the addition of emulsion and initiator solution, the system continued to react for 3 h at 85 °C. Then the temperature of the whole system was reduced to below 40 °C.

### 4.3. Preparation of Polyacrylate Coated Urea

The fertilizer granules were coated in a Wurster fluidized-bed equipped with a bottom-spray pneumatic nozzle (LDP-3, Changzhou Jiafa Granulation Drying Equipment Co., Ltd., Changzhou, China). The product temperature was set to 45–50 °C; the spray rate was set at 2.5 g/min; the atomization pressure was set to 0.1 MPa. After the coating process, the granular coated urea was dried in a vacuum rotary evaporator and about 0.1% talcum powder was added to prevent film adhesion among urea granules. Three different coating rates of PACU were finally prepared, and the dry matter of the emulsion accounted for 3%, 5%, and 8% of the weight of urea granules, respectively. To be closer to the actual production, PACU was prepared in the factory environment.

### 4.4. Preparation of CW Modified PACU

The PACU was modified by CW in a rotary evaporator (RE-52, Shanghai Yarong Biochemical Instrument Factory, Shanghai, China). The PACU and CW were accurately weighed and mixed into the round-bottom flask. The flask was rotated to ensure that the PACU and CW were mixed well and evenly and then was heated in a water bath. After that, the flask was cooled down to room temperature and the CW was loaded onto the surface of the PACU. The effects of heating temperature, the ratio of PACU and CW, the rotation speed, and the rotation time on the loading amount of CW were investigated. Finally, the PACU with CW loading amounts of 0.0% 0.3%, 0.5%, 0.8%, and 1.0% were produced and a total of 15 different coated urea samples were obtained.

### 4.5. Characterization of the Coating Films

The scanning electron microscope (SEM) was used to observe the surface and section characteristics of the fertilizers. The PACU and WCPACU with different wax amounts were selected, cut, and fixed on the support. The surface and section morphology were scanned using a JCM 6000 NeoScope benchtop SEM (JEOL, Peabody, MA, USA). The surface functional group of the PACU and WCPACU were characterized by a handheld TruDefender Fourier transform spectrometer (TRUDEFENDER FT, Thermo Scientific, Waltham, MA, USA). The coating film on PACU and CWPACU was torn off and then pressed on the attenuated total reflectance (ATR) crystal. Attenuated total reflection Fourier transform infrared spectroscopy (FTIR-ATR) of the unmodified and modified coating were acquired in the range of 4000–650 cm^−1^ with a spectral resolution of 4 cm^−1^. Each spectrum was finally recorded by averaging 64 successive scans. The background spectrum was scanned before each sample scanning to correct the atmospheric interference and the instrumental noise. The pure carnauba wax also was characterized using the same process. The element contents in coatings before and after biomimetic modification were in-situ characterized by a handheld Laser-induced breakdown spectrometer (Z-300 LIBS Analyzer, SciAps, Woburn, MA, USA). The granular PACU and CWPACU fertilizers were directly placed onto the detection window and shots with 4 × 4 matrices were applied for each sample. The frequency, delivery energy, and wavelength of the pulsed laser were set as 5 mJ per pulse, 50 Hz, and 1064 nm, respectively. Argon was applied as ambient gas during spectra acquisition to exclude the interference of air. The Laser-induced breakdown spectroscopy (LIBS) of the coatings with a wavelength from 190 nm to 950 nm and a spectral resolution of 0.1 nm were acquired.

### 4.6. Spectral Preprocessing and Data Analysis

The FTIR-ATR and LIBS spectra were smoothed by wavelet transform and normalized before spectral analysis in MATLAB R2020b (The Math Works, Natick, MA, USA). Principal component analysis (PCA) was performed to illustrate the internal structure of spectra.

### 4.7. Nutrient Release Profile

Five grams of fertilizer were accurately weighed and placed in a glass bottle containing 100 mL of deionized water. The sample was then cultured at 25 °C in a constant temperature incubator. The solutions were sampled and exchanged with 100 mL of deionized water on days 1, 3, 5, 8, 11, 14, 17, 21, 25, 29, 34, 39, 51, and 70, respectively. The solution urea concentration was measured by an FTIR spectrometer (Nicolet 6700, Thermo Scientific, Waltham, MA, USA) equipped with a ZnSe crystal [49]. Briefly, a series of referenced urea solutions with a concentration of 0, 0.5, 1, 5, 10, 20, 30, 40, and 50 g L^−1^ were prepared using deionized water. The FTIR spectra of referenced urea solutions were obtained by averaging 32 successive scans under a moving mirror velocity of 0.32 cm s^−1^. The FTIR spectra were then smoothed by wavelet transform in MATLAB R2020b. The stretching vibration of C=O at 1750–1340 cm^−1^ (peak area) was applied to build the calibration curve for urea concentration. The urea concentrations (c) in released solutions were determined according to the calibration curve. The release rate of urea was calculated by the following formula:

$$R=\frac{c\times V\times 10^{-3}}{m\times \rho }\times 100\%$$

where *c* is the measured urea concentration in solution (g L^−1^), *V* is the volume of released solution (mL), *m* is the weight of coated urea (g), and *ρ* is the urea content of the coated urea (%).

## 5. Conclusions

A biomimetic modification of water-based polymer-coated fertilizers by CW was reached through simulating the structure and components of plant leaf surface layer. As compared with pure PACU, CW modified PACU had much longer release longevity, and their release curves had a more pronounced connection with the plant’s nutrients demand; due to the natural superhydrophobicity of CW, it effectively improved the contact between the fertilizer core and water. Covering the original polyacrylate membrane with biological wax changed the pore size in the coating, and delayed the exchange of nutrients across the coated membrane. Additionally, CW modification saved coating material of polyacrylate, which reduced the production costs. Therefore, the novel biomimetic modification by biological wax showed a broad application potential in the industry of coated controlled release fertilizer.

## Data Availability

Not applicable.
